# Robotic Repair of a Missed Traumatic Diaphragmatic Injury in a Prior Gastric Bypass Patient With Herniation of the Gastric Remnant

**DOI:** 10.7759/cureus.76785

**Published:** 2025-01-02

**Authors:** Madelyn N Ross, Adolfo A Torres, Jamie Anderson, Semeret Munie, Jennalee Corsello, Darren B Nease

**Affiliations:** 1 Medical Education, Marshall University Joan C. Edwards School of Medicine, Huntington, USA; 2 General Surgery, Marshall University Joan C. Edwards School of Medicine, Huntington, USA; 3 Bariatric Surgery, Marshall University Joan C. Edwards School of Medicine, Huntington, USA

**Keywords:** abdominal blunt trauma, bariatric case report, motor vehicle accident, robotic diaphragm hernia repair, roux-en y, traumatic diaphragmatic hernia

## Abstract

Traumatic diaphragmatic injuries following blunt or penetrating trauma have a low incidence rate. Symptoms can be obscured by distracting injuries or, in some cases, patients can be asymptomatic, making diagnosis difficult.

A 57-year-old female who underwent a robotic Roux-en-Y gastric bypass with a hiatal hernia repair in 2021, presented as an alerted trauma in February 2024 following a motor vehicle accident (MVA). She was an unrestrained driver traveling 35 mph with airbag deployment. During an assessment, it was noted that she had extensive left breast and chest ecchymosis; however, trauma scans were negative. She was admitted for observation and was discharged the next day.

Three weeks following the accident, she presented with dull epigastric pain and early satiety, which progressed to dysphagia to solids and significant reflux. Her initial CT scan was concerning a recurrent hiatal hernia. A nasogastric tube was placed and repeat imaging with oral contrast revealed that her gastric remnant had herniated into her chest. Given her recent trauma, there was concern for a missed diaphragmatic injury.

The patient was admitted, and the decision was made to perform a robotic reduction and repair of the diaphragmatic hernia on hospital day 3. Intraoperatively, it was noted that her previous hiatal hernia repair was intact and there was a large traumatic defect in the 3 o’clock position of the left hiatus. The excluded gastric remnant and omentum were reduced back into the abdomen and the diaphragmatic defect was repaired primarily. The gastric remnant had multiple areas concerning for devascularization and a subtotal gastric resection was performed. The patient did well after surgery and was discharged home the next day. She regularly follows up with the bariatric clinic and reported doing well with no complaints at her six-month follow-up.

This case shows the significance of missed diaphragmatic injuries in trauma, the value of having a high clinical suspicion for injury, and the added complexity of this being a bariatric patient with a previous gastric bypass. It also highlights the successful outcome of a robotic repair.

## Introduction

Diaphragmatic injuries, though relatively rare, pose significant diagnostic and management challenges. The incidence of these injuries following blunt or penetrating trauma ranges from 0.8% to 5%, with a notable percentage (up to 30%) experiencing delayed diagnosis [1]. Such injuries can be categorized into three distinct phases: the initial phase occurring at the time of injury, the latent phase characterized by transient herniation, and the obstructive phase [2]. Most injuries are found on the left side of the diaphragm due to the barrier effect of the liver on the right side [3].

Despite the known phases and mechanisms, diagnosis can be complicated by the presence of distracting injuries or asymptomatic presentations. The initial diagnosis in this case may have been delayed due to the emergent situation of the patient’s initial presentation. In a trauma setting, abdominal CT imaging is not performed with oral contrast because it cannot be administered in a timely manner and thus would cause undue delay in diagnosing life-threatening injuries. Additionally, the patient's history of a previous hiatal hernia repair may have been a confounding factor in the interpretation of the subsequent CT scan on readmission. Early and accurate diagnosis is crucial, as delayed treatment may result in morbidities such as intestinal obstruction, pneumopericarditis, and tension faeco-pneumothorax [1].

This type of traumatic injury can pose additional challenges in bariatric patients and add to the complexity of the case. In a recent case report, a 45-year-old woman with a history of laparoscopic Roux-en-Y gastric bypass experienced incarceration of the gastric remnant [4]. She presented four years post-operation with left chest pain that had been ongoing for two weeks and acutely worsened two days before her visit. CT scan confirmed that the remnant stomach was in the left chest [4]. She was initially taken to the operating room for laparoscopic repair; however, the procedure was converted to an open approach due to difficulties in reducing the remnant laparoscopically [4].

This case report highlights a similar presentation of this rare instance of a delayed diagnosis of a traumatic diaphragmatic injury (TDI) in a patient who had a history of a robotic Roux-en-Y gastric bypass with hiatal hernia repair three years prior. The patient’s initial trauma evaluation was unremarkable, but subsequent symptoms and imaging revealed a significant diaphragmatic defect. This case shows the importance of considering diaphragmatic injury in trauma patients with gastrointestinal symptoms, even when initial imaging does not reveal an obvious injury. The objective of this report is to illustrate the diagnostic and management challenges associated with traumatic diaphragmatic injuries and emphasize the successful outcome of this robotic repair.

## Case presentation

Patient information

A 57-year-old female, three years postoperative from a robotic Roux-en-Y gastric bypass and hiatal hernia repair for morbid obesity with a BMI of 52, presented following a motor vehicle accident in February 2024. She was an unrestrained driver traveling at 35 mph with airbag deployment.

Clinical findings and diagnostics

**Figure 1:**
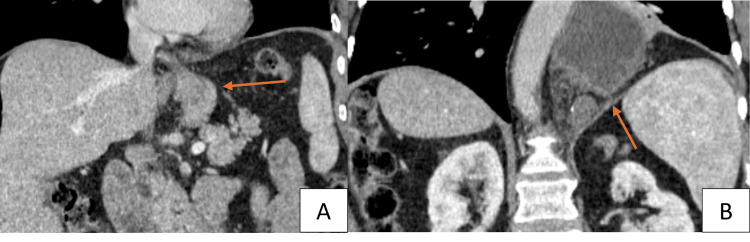
Pouch below the diaphragm (A), gastric remnant in the chest (B)

During assessment, it was noted that the patient had extensive left breast and chest ecchymosis; however, a CT scan of the abdomen and pelvis with IV contrast came back negative. She was admitted for observation and then discharged the following day. Three weeks later, she presented to the emergency department with dull epigastric pain and early satiety that started one week after the motor vehicle accident (MVA), which further progressed to dysphagia to solids and significant reflux over the next two weeks. Her initial CT scan was concerning a recurrent hiatal hernia. Further imaging with oral contrast revealed herniation of the gastric remnant into the chest (Figure 1). Given her recent trauma, there was concern for a missed diaphragmatic injury.

Management

**Figure 2:**
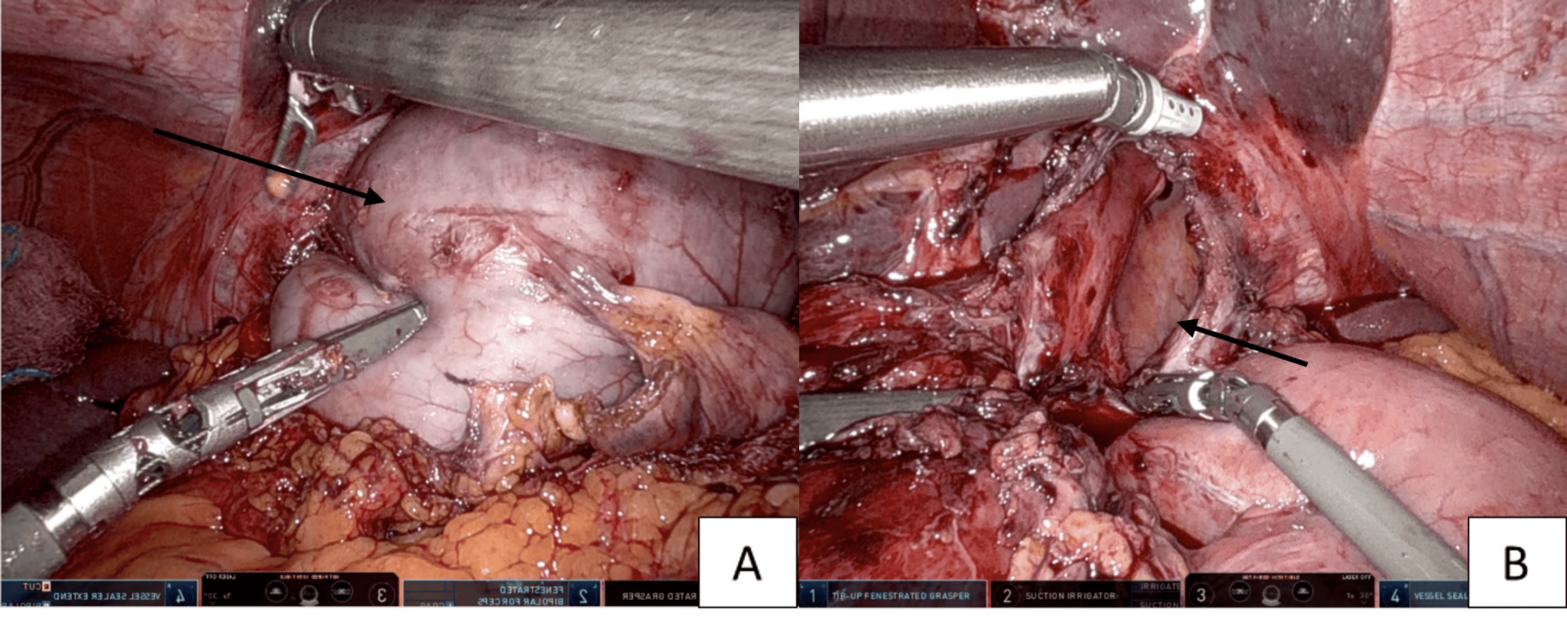
Reduction of the gastric remnant (A), traumatic diaphragmatic injury after reduction of the gastric remnant and omentum (B)

The patient was admitted, and the decision was made to perform a robotic reduction and repair of the diaphragmatic hernia on hospital day 3. Intraoperatively, it was noted that her previous hiatal hernia repair was intact and there was a large traumatic defect in the 3 o’clock position of the left hiatus (Figure 2, panel B). The excluded gastric remnant and omentum were reduced back into the abdomen (Figure 2, panel A) and the diaphragmatic defect was repaired primarily. The gastric remnant had multiple areas concerning for devascularization and a subtotal gastric resection was performed.

Outcome and follow-up

Postoperatively, the patient recovered well and was discharged the following day. She continues to do well with no reported complications at her six-month follow-up visit.

## Discussion

Delayed presentation 

In this case, the patient presented three weeks after an MVA with symptoms of epigastric pain, early satiety, dysphagia, and reflux. Initial trauma imaging was negative, but the repeat CT on subsequent presentation, suggested a recurrent hiatal hernia. However, there was high clinical suspicion of a missed diaphragmatic injury that had allowed for the subsequent herniation. This prompted further imaging that revealed herniation of the gastric remnant into the chest. Traumatic diaphragmatic injuries are often missed initially, with symptoms sometimes appearing days or even years after trauma [1]. As many as 30% of TDIs may present delayed [1]. For instance, this patient's symptoms emerged weeks post-trauma, consistent with the variability in presentation reported in previous research [1].

Mechanism of injury

High-velocity trauma can lead to diaphragmatic rupture due to a sudden increase in intra-abdominal pressure, causing the diaphragm to tear and allow for herniation to occur [1]. Traumatic diaphragmatic injuries are classified into blunt and penetrating types based on the mechanism of injury. This distinction is significant for understanding associated injuries, complications, and overall mortality rates. Penetrating trauma accounts for two-thirds of TDIs while the remaining one-third are caused by blunt trauma [5]. Specific to motor vehicle collisions, TDI occurs in 1-5% of motor vehicle collisions [6].

Diagnostic imaging

Historically, diaphragmatic injuries were often missed due to limitations in imaging techniques, with preoperative detection rates ranging from 12-69% [1]. CT scans are currently the gold standard for diagnosing traumatic diaphragmatic injuries from blunt trauma, with an 80% sensitivity and 98% specificity [7]. This case highlights the importance of considering diaphragmatic injury in trauma patients with persistent or new symptoms, even if initial scans are negative, as repeat imaging was crucial for detecting this patient’s diaphragmatic injury.

The guidelines put forth by the Easter Association for the Surgery of Trauma (EAST) in 2018 for the management of TDIs compared the sensitivity and specificity of CT imaging to diagnostic laparoscopy for diagnosis. As expected, laparoscopy missed fewer injuries but was associated with increased risk [8]. This resulted in a conditional recommendation for laparoscopy over CT in the setting of thoracoabdominal stab wounds for suspected TDIs [8]. However, this recommendation does not apply to blunt mechanisms, like in this case.

Surgical and repair approach

Current studies examining the surgical technique in multiple cases of traumatic diaphragmatic hernias showed a predominance of the abdominal approach for repair. It is difficult to assess which approach provides the greatest benefit to morbidity and mortality due to low-quality evidence and the lack of patient-matched studies that compare the abdominal and thoracic approaches [8]. In the absence of clear guidelines, the initial approach to the repair of a traumatic diaphragmatic hernia should be chosen based on the abilities and preferences of the surgeon. The presence of secondary visceral injury associated with the hernia should help guide the surgeon toward one approach or the other [8]. For instance, an abdominal approach would be preferable in the setting of hernia incarceration or concern for hollow organ perforation, but a thoracic approach may be required if there is concern for lung or cardiac injury.

The efficacy of using mesh in the closure of traumatic diaphragmatic injuries as opposed to closing primarily with sutures is currently unclear. This is due to, in part, the rarity of TDIs and the high variability of their presentation, which transfers to the lack of peer-reviewed studies including patients with this pathology. Studies involving the use of mesh for the repair of paraesophageal and hiatal hernias have found short-term benefits for their use (Level 1B) [9]. There is no current analysis of whether the use of mesh in these cases provides long-term benefits [9]. There are limited recommendations available regarding the use of mesh for traumatic diaphragmatic hernia repair. Both primary closure and mesh augmentation carry a class 2C recommendation due to low-quality evidence [6].

The decision to perform hiatal hernia repairs laparoscopically or robotically primarily depends on surgeon preference and training. A recent study in 2021 examined the short-term benefits of each surgical approach and found that there was a steady increase in the utilization of robotic devices for diaphragmatic hernia repairs [10]. They also determined that the robotic approach was associated with the highest index hospitalization costs compared to laparoscopic and open approaches [10]. Laparoscopic diaphragmatic hernia repair was associated with the lowest cost and shortest length of stay, but the short-term clinical outcomes of laparoscopic and robotic repairs remained similar [10].

## Conclusions

This case is unique in that it involves the added complexity of a previous hiatal hernia repair and Roux-en-Y gastric bypass. Because of the rarity of this clinical scenario, this case represents the first documented successful robotic repair of a delayed traumatic diaphragmatic injury with an incarcerated gastric remnant. It emphasizes the importance of recognizing overlooked diaphragmatic injuries in trauma patients and the value of having a high clinical suspicion for injury. Additionally, it exemplifies the unique challenges specific to bariatric patients due to the anatomical alterations after a gastric bypass. Lastly, it highlights the successful outcome of robotic repair, emphasizing the importance of innovative surgical techniques in managing complex cases.
